# Genetic diversity and population structure analysis of Ghanaian and exotic cassava accessions using simple sequence repeat (SSR) markers

**DOI:** 10.1016/j.heliyon.2019.e03154

**Published:** 2020-01-31

**Authors:** Joseph Adjebeng-Danquah, Joseph Manu-Aduening, Isaac Kwadwo Asante, Richard Yaw Agyare, Vernon Gracen, Samuel Kwame Offei

**Affiliations:** a CSIR-Savanna Agricultural Research Institute, P. O. Box TL 52, Tamale, Ghana; b CSIR-Crops Research Institute, Fumesua, P.O. Box 3785, Kumasi, Ghana; c West Africa Centre Crop Improvement (WACCI), University of Ghana, Legon, Accra, Ghana

**Keywords:** Agriculture, Environmental science, Molecular markers, Genetic variability, *Manihot esculenta* Crantz, Heterozygosity

## Abstract

Genetic diversity is fundamentally important in crop improvement and provides plants with the capacity to meet the demands of changing environments. This work was carried out to assess the diversity and the extent of genetic relatedness among a number of assembled cassava (*Manihot esculenta* Crantz) accessions. We conducted a microsatellite marker analysis of 89 cassava accessions collected from Ghanaian and exotic sources. These accessions were assayed using 35 simple sequence repeat (SSR) markers. A total of 167 alleles were detected from 35 polymorphic markers with an average of 4.77 alleles per locus. High allelic frequency was detected across the accessions, ranging from 0.32 to 0.99 with an average of 0.62 per marker. Observed heterozygosity ranged from 0.03 - 0.97 across the accessions. Polymorphism information content (PIC) ranged from 0.03 to 0.78 with a mean of 0.45, indicating high level of polymorphism across the accessions. Comparatively, higher number of alleles, gene diversity and observed heterozygosity were detected among the local accessions compared with the exotic accessions indicating rich genetic diversity among them. Population structure analysis based on STRUCTURE identified two subpopulations and a large number of admixtures. Cluster analysis based on the neighbour joining algorithim further separated the collection into seven sub-groupings irrespective of geographical origin. This indicates the possible sharing of common genomic regions occurring across the accessions. High allelic frequency differences and levels of heterozygosity were observed among the germplasm. These findings indicated significant genetic variability in the germplasm to warrant selection.

## Teaser

Agriculture, Environmental Science; Molecular markers; genetic variability; *Manihot esculenta* Crantz; Heterozygosity; Genetic variability**.**

## 1 Introduction

Cassava (*Manihot esculenta* Crantz) is an important staple crop, widely cultivated and consumed in Sub-Saharan Africa. It is mostly grown by smallholder farmers often in marginal ecologies due to its ability to give better and appreciable yields than most staple crops in ecologies of drought and poor soils [1, 2]. The crop is fast gaining popularity as an important industrial raw material in Ghana, leading to its widespread cultivation particularly in the savannah ecologies [3, 4]. However average yields at farm level are low (8t ha^−1^) [5], compared with a potential yield of 90 t/ha under good agronomic management [2]. This could be due to the use of low yielding varieties that are susceptible to pests and diseases, as well as high sensitivity to harsh environmental conditions [6]. Thus, there is the need to develop new improved varieties that are adapted to these environments.

Progress made in a breeding programme depends on a better understanding of the genetic variability present in the population assembled [7]. In some cases, the genetic base is broadened through hybridisation with wild and related species [8], plant introductions from external sources or locally assembled germplasm [9, 10]. Local germplasm, particularly adapted landraces from farmers’ fields, are valuable genetic resources for crop improvement [11, 12]. Significant genetic variability has been reported across the cassava gene pool for traits associated with tolerance to hash environments (such as drought) [2, 13] which can be exploited.

Several studies have reported the existence of diverse germplasm of crops on farmers fields as a result of *in situ* germplasm conservation and farmer to farmer planting materials transfer [14]. Farmers keep a wide range of crop varieties to provide harvest security, yield stability and the possibility to adapt to changing ecological conditions [15, 16]. Through the exchange of planting materials among farmers [17], accessions are given unique names at their new location [18]. The same genetic materials are often given different names resulting in duplicates when germplasm are assembled from such localities [19, 20, 21, 22]. This results in underestimation or overestimation of the actual genetic diversity present in the population [23].

Ability to remove duplicates during characterization of germplasm collections for breeding activities is very important. Characterization of crops can be done using agronomic, phenotypic descriptors or molecular markers [7, 24]. Phenotypic characterization is easy, rapid to score, and provides useful preliminary information for diversity assessment of germplasm [5, 24, 25]. Phenotypic descriptors have been used to effectively characterise several crop genotypes, particularly in stressful environments [5, 26, 27, 28]. However, most phenotypic descriptors particularly quantitative characters, are not very reliable as markers due to strong influence by genotype - environment interaction [29]. For this reason, analysis of diversity at the molecular level is used to validate and/or complement phenotypic characterization [5, 30].

Molecular markers are segments of DNA that can represent different functional classes and have been widely used to estimate genetic variation among different populations [31, 32]. An ideal molecular marker must be highly heritable, applicable to any part of the genome and polymorphic enough to enable the discrimination of closely related genotypes [31, 33]. Molecular markers are easily detectable and stable in plant tissues irrespective of environmental influence [5, 32, 33]. Available molecular markers for assessing genetic diversity include random amplified polymorphic DNA (RAPD) [34]; amplified fragment length polymorphism (AFLP) [33]; allozymes [35], simple sequence repeats (SSRs) [36]; single nucleotide polymorphism markers (SNPs) [37, 38] and diversity array technology markers (DArT) [39]. Specifically, SSRs are the markers of choice for genetic diversity analysis due to their abundance in the genome, highly polymorphism and codominant nature, which make them useful for characterizing heterozygote plants such as cassava [36]. SSR markers have been successfully used to assess genetic diversity between cassava accessions and their wild relatives [5, 40]. The objective of this study was to characterize and assess the extent of genetic diversity among 89 cassava accessions collected from Ghanaian and exotic sources using simple sequence repeat (SSR) markers.

## 2 Materials and methods

### 2.1 Plant materials

The study involved 89 cassava accessions obtained mainly from farmers’ field in Ghana, CSIR-Crops Research Institute (Kumasi, Ghana), International Centre for Tropical Agriculture (Cali, Colombia), CSIR-Savanna Agricultural Research Institute (Nyankpala, Ghana) and International Institute of Tropical Agriculture (Ibadan, Nigeria). Names of the various accessions and the place of collection are presented in Table 1.

**Table 1:** List of cassava accessions used for the study.

No.	Accessions	Source	No.	Accessions	Source	No.	Accessions	Source
1	AFS2000/023	Local	31	Nkoranza	Local	61	97/4769	IITA
2	AFS2000/043	Local	32	SAA 007	Local	62	98/0505	IITA
3	AFS2000/131	Local	33	SAA 004	Local	63	98/0581	IITA
4	ATR002	Local	34	TA97/054	Local	64	98/2226	IITA
5	ATR007	Local	35	UCC2001/449	Local	65	99/0240	IITA
6	Bankyebrodie	Local	36	UCC2001/464	Local	66	99/0554	IITA
7	BD96/009	Local	37	Kwasiabedi	Local	67	00/0093	IITA
8	BD96/021	Local	38	00/0140	IITA	68	I91934	IITA
9	BD96/040	Local	39	00/0203	IITA	69	MM 96/JW1	IITA
10	BD96/093	Local	40	00/0338	IITA	70	MM 96/1751	IITA
11	BD96/154	Local	41	00/0354	IITA	71	TME 419	IITA
12	TA97/137	Local	42	00/0364	IITA	72	TME 435	IITA
13	UCC2001/104	Local	43	01/0046	IITA	73	TME 693	IITA
14	UCC2001/111	Local	44	01/0069	IITA	74	96/0067	IITA
15	BAN 001	Local	45	01/0093	IITA	75	96/1642	IITA
16	AWA 004	Local	46	01/0114	IITA	76	97/0783	IITA
17	BIABASSE ∗	Local	47	01/0134	IITA	77	97/0879	IITA
18	Debor	Local	48	01/0169	IITA	78	CTSIA1	CIAT
19	DMA 005	Local	49	01/0220	IITA	79	CTSIA110	CIAT
20	Essiabayaa	Local	50	01/1088	IITA	80	CTSIA112	CIAT
21	KSI2000/092	Local	51	01/1412	IITA	81	CTSIA131	CIAT
22	KSI2000/126	Local	52	02/0540	IITA	82	CTSIA133	CIAT
23	KSI2000/191	Local	53	191/02324	IITA	83	CTSIA162	CIAT
24	KW2000/53	Local	54	2000/0388	IITA	84	CTSIA230	CIAT
25	Kwanwoma	Local	55	94/0006	IITA	85	CTSIA45	CIAT
26	NWA 004	Local	56	96/0708	IITA	86	CTSIA48	CIAT
27	OFF2000/019	Local	57	96/1708	IITA	87	CTSIA65	CIAT
28	OFF2000/023	Local	58	96/409	IITA	88	CTSIA72	CIAT
29	OFF2000/145	Local	59	97/1856	IITA	89	CTSIA8	CIAT
30	Ponti∗	Local	60	98/2132	IITA			

∗ Farmer preferred varieties, Local = accessions collected locally from farmers' field and research stations in Ghana, IITA = accessions obtained from International Institute of Tropical Agriculture (IITA), CIAT = International Centre for Tropical Agriculture (CIAT).

### 2.2 Simple sequence repeat marker analysis

#### 2.2.1 Genomic DNA extraction

DNA extraction was done at the Biotechnology Laboratory of CSIR-Savanna Agricultural Research Institute (SARI), Nyankpala. Genomic DNA was extracted from the 89 cassava accessions using CTAB method [41] with slight modifications. Leaves were sampled from two weeks old cassava cuttings which were raised in pots for the DNA extractions. About 20 mg of the leaf sample from each plant was taken and ground in 2.0 ml Eppendorf tubes into fine powder with liquid nitrogen. Then 800 μl of 2% CTAB and 0.5 μl of 0.1% mercaptoethanol were added. The samples were incubated in a sand bath at 65 °C for 30 min with intermittent vortexing. The samples were cooled at room temperature after which equal volumes (800 μl) of chloroform isoamyl alcohol (24:1) were added. The tubes were inverted several times to ensure that a thorough mixture was obtained and then centrifuged at 14000 rpm for 15 min. Equal volumes of the chloroform isoamyl alcohol solution were added to the samples in clean 1.5 ml Eppendorf tubes and centrifuged at 14000 rpm for 15 min. Nucleic acids were precipitated by adding two thirds volume of ice cold isopropanol (400 μl) whilst shaking gently. Precipitation was enhanced by storing the samples at -20 °C overnight. Pelleting of nucleic acids was done by centrifuging at 14000 rpm for 5 min. The isopropanol was decanted and the pellet was washed with 500 μl of washing buffer. The washing buffer was decanted and the pellet was washed in 400 μl of ethanol (80%) and then centrifuged at 6000 rpm for 4 min. The ethanol was decanted and the pellet was dried. The DNA was suspended in 100 μl of TE buffer and centrifuged at high speed for 30 s and stored at 4 °C until ready for use. DNA of each accession was confirmed by electrophoresis on 2% agarose gel stained with ethidium bromide (3 μl) which revealed positive results.

#### 2.2.2 SSR (microsatellite) markers and PCR amplification

A total of 35 simple sequence repeat (SSR) primers, widely distributed across the cassava genome [42], were used for the study (Table 2). The SSR markers were synthesized at Metabion International AG (Germany). Polymerase chain reactions (PCR) were carried out in a Techne Thermalcycler (TC- 412) in a 10 μl reaction mixture in 96-well plates. PCR master mix kits (KAPA 2G Fast ReadyMix with dye) procured from KAPA Biosystems (Pty) Ltd (South Africa) were used for the amplification. The kit 2X PCR master mix contained KAPA2G Fast DNA Polymerase (0.2 U per 10 μl reaction), KAPA2 Fast PCR buffer, dNTPs (0.2 mM each at 1X), MgCl_2_ (1.5 mM at 1X), stabilizers and loading dye. An amount of 1 μl of genomic DNA and 0.5 μl each of forward and reverse primers were added to the PCR kits for DNA amplification. PCR amplifications were done with the following conditions: initial denaturation at 95 °C for 3 min, denaturing at 95 °C for 10 s, annealing at X^0^C (annealing temperatures for each marker, Table 2) for 10 s and extension at 72 °C for 10 s. The reaction was repeated for 35 cycles and a final extension at 72 °C for 10 min was carried out. The reactions were then held at 4 °C until electrophoresis.

**Table 2:** Simple sequence repeat (SSR) primers used for the study.

No	Primer name	Sequence (5′-3′)		Ta (^0^C)
		Forward primer	Reverse primer	
1	NS 189	TGGGCTGTTCGTGATCCTTA	CATGAGTTTAAAAATTATCACATCCG	54
2	NS 376	TCAAGACCCTTGCTTTGGTT	GGACTATCAAGGCGCAAAAG	52
3	SSRY 4	TGAGAAGGAAACTGCTTGCAC	CAGCAAGACCATCACCAGTTT	55
4	SSRY 5	GGAAACTGCTTGCACAAAGA	CAGCAAGACCATCACCAGTTT	51
5	SSRY 9	AACTGTCAAACCATTCTACTTGC	GCCAGCAAGGTTTGCTACAT	53
6	SSRY 12	TCACCGTTAATTGTAGTCTGCG	GCGAGGTTCAAATATGCGAT	54
7	SSRY 19	CCAGAAACTGAAATGCATCG	AACATGTGCGACAGTGATTG	53
8	SSRY 20	GTACATCACCACCAACGGGC	AGAGCGGTGGGGCGAAGAGC	54
9	SSRY 21	GGCTTCATCATGGAAAAACC	CAATGCTTTACGGAAGAGCC	52
10	SSRY 34	AGTGGAAATAAGCCATGTGATG	CCCATAATTGATGCCAGGTT	52
11	SSRY 45	CGTTGATAAAGTGGAAAGAGCA	ACTCCACTCCCGATGCTCGC	53
12	SSRY 48	AAGGAACACCTCTCCTAGAATCA	CCAGCTGTATGTTGAGTGAGC	51
13	SSRY 50	TCAAACAAGAATTAGCAGAACTGG	TGAGATTTCGTAATATTCATTTCACTT	54
14	SSRY 59	ACAGCTCTAAAAACTGCAGCC	AACGTAGGCCCTAACTAACCC	52
15	SSRY 63	TGACTAGCAGACACGGTTTCA	GCTAACAGTCCAATAACGATAAGG	52
16	SSRY 64	ACCACAAACATAGGCACGAG	CACCCAATTCACCAATTACCA	59
17	SSRY 69	CCTTGGCAGAGATGAATTAGAG	GGGGCATTCTACATGATCAATAA	54
18	SSRY 78	GGTAGATCTGGATCGAGGAGG	CAATCGAAACCGACGATACA	53
19	SSRY 82	GGAATTCTTTGCTTATGATGCC	TTCCTTTACAATTCTGGACGC	53
20	SSRY 103	TGTAAGGCATTCCAAGAATTATCA	TCTCCTGTGAAAAGTGCATGA	54
21	SSRY 106	CATTGGACTTCCTACAAATATGAAT	TGATGGAAAGTGGTTATGTCCTT	52
22	SSRY 120	CCTGCCACAATATTGAAATGG	CAACAATTGGACTAAGCAGCA	53
23	SSRY 135	TTCCAGACCTGTTCCACCAT	ATTGCAGGGATTATTGCTCG	51
24	SSRY 147	ATAGAGCAGAAGTGCAGGCG	CTAACGCACACGACTACGGA	60
25	SSRY 148	TGAAACTGTTTGCAAATTACGA	TCCAGTTCACATGTAGTTGGCT	52
26	SSRY 151	TGAAAATCTCACTGGCATTATTT	TCATAAAGCTCGTGATTTCCA	52
27	SSRY 155	TGATGAAATTCAAAGCACCA	CGCCTACCACTGCCATAAAC	57
28	SSRY 161	CCGCTTAACTCCTTGCTGTC	CAAGTGGATGAGCTACGCAA	56
29	SSRY 164	GCAATGCAGTGAACCATCTTT	CGTTTGTCCTTTCTGATGTTC	55
30	SSRY 169	TCAGAATCATCTACCTTGGCA	AAGACAATCATTTTGTGCTCCA	55
31	SSRY 175	CGACAAGTCGTATATGTAGTATTCACG	GCAGAGGTGGCTAACGAGAC	56
32	SSRY 177	CGATCTCAGTCGATACCCAAG	CACTCCGTTGCAGGCATTA	53
33	SSRY 180	TGCACACGTTCTGTTTCCAT	ATGCCTCCACGTCCAGATAC	55
34	SSRY 181	TGTGACAATTTTCAGATAGCTTCA	CACCATCGGCATTAAACTTTG	55
35	SSRY 182	ACAATTCATCATGAGTCATCAACT	CCGTTATTGTTCCTGGTCCT	53

Ta (^0^C) = Annealing temperature. Sources of SSR markers [40, 42].

#### 2.2.3 Gel electrophoresis

Gel electrophoresis were carried out in Polyacrylamide gel (6%) using Galileo Bioscience (81–2325) horizontal tank, in 100ml 1X TE running buffer stained with ethidium bromide (3 μl). Electrophoresis was carried out at 120V for 150 min using 10 μl of the amplified PCR products. Then 50 bp and 100 bp molecular ladder (Ladder Plus) obtained from NBS Biologicals Ltd (Cambridge, UK) were used to estimate the molecular weight of the amplified products. The PCR products were visualized and photographed on Benchtop UV trans-illuminator.

### 2.3 Data analysis

The DNA bands were scored based on the fragment length of each allele. The assignment of the fragment was based on its position relative to the 50 and the 100 bp standard molecular marker (DNA Ladder) used. Alleles were scored as present (1) or absent (0). Band sizes for each marker per genotype were scored as a/b where ‘a’ is the upper band and ‘b’ is the lower band. PowerMarker version 3.25 [43] was subsequently used to detect allele frequency, allele number per locus, gene diversity, observed heterozygosity and polymorphism information content (PIC) for each marker across all the 89 accessions. Analysis of molecular variance (AMOVA) was performed to distinguish the molecular genetic variance within and among populations using GenAlEx6.502 software [44]. For the population structure analysis, the data from the 35 polymorphic SSR markers was imperiled to population structure analysis based on the admixture model clustering method in the software package STRUCTURE 2.3.4 [45]. This model was run by varying the number of assumed population (K) from 1 to 12 with 5 alterations for each K. A burn-in period of 10 000 and Markov Chain Monte Carlo (MCMC) replications of 20,000 after each burn-in was used. The optimum population (K) which best estimated the structure of the 89 accessions was predicted using the Evanno's method [46] through the online based software STRUCTURE HARVESTER [47]. The model was repeated for the K at maximum ΔK with a burn-in period of 100,000 and an MCMC of 200,000 after each burn-in with one alteration. The accessions were assigned to each subpopulation based on their probability of association of ≥60% to each of the two groups, accessions with probability of association <60% were considered as admixtures. Genotypic associations (cluster analysis) were analysed in DARwin 5 [48] using the simple matching coefficient and neighbour-joining algorithim. To ensure reliability of the results, 10,000 bootstraps were performed in the construction of the dendrogram.

## 3 Results

### 3.1 Allelic diversity

A total of 167 alleles were generated by the 35 SSR markers (Table 3). Allele frequency ranged from 0.32 to 0.99 for SSRY-164 and SSRY-48, respectively, with a mean of 0.62. SSRY-169 had the highest number of polymorphic bands (86) whilst SSRY-120 had the lowest (9) among the genotypes. Allele number per locus ranged from 2 to 10 with a mean of 4.77 alleles per locus. Gene diversity varied from 0.03 to 0.81, with primers SSRY-48 and SSRY-164 having the lowest and highest gene diversity, respectively. Primer SSRY-180 had the highest observed heterozygosity of 0.97 while primer SSRY-48 had the lowest of 0.03. Polymorphism Information Content (PIC) ranged from 0.03 to 0.78 with a mean of 0.45. Primer SSRY-164 was the most polymorphic with a PIC value of 0.78.

**Table 3:** Results of the genetic diversity parameters for each of the 35 SSR loci analysed across 89 cassava accessions.

Marker	Allele frequency	No. of polymorphic bands	Allele number per locus	Gene diversity	H_o_	PIC
NS-189	0.67	41.00	5.00	0.49	0.24	0.43
NS-376	0.66	54.00	5.00	0.52	0.50	0.48
SSRY-4	0.68	39.00	3.00	0.47	0.54	0.40
SSRY-5	0.45	22.00	3.00	0.59	0.36	0.51
SSRY-9	0.33	18.00	6.00	0.76	0.44	0.72
SSRY-12	0.52	68.00	5.00	0.53	0.24	0.43
SSRY-19	0.34	51.00	6.00	0.76	0.59	0.72
SSRY-20	0.70	48.00	7.00	0.49	0.35	0.47
SSRY-21	0.60	56.00	4.00	0.57	0.48	0.51
SSRY-34	0.92	75.00	3.00	0.15	0.11	0.14
SSRY-45	0.57	57.00	5.00	0.57	0.75	0.51
SSRY-48	0.99	70.00	3.00	0.03	0.03	0.03
SSRY-50	0.60	71.00	6.00	0.59	0.30	0.54
SSRY-59	0.47	38.00	5.00	0.57	0.18	0.48
SSRY-63	0.75	69.00	5.00	0.42	0.10	0.40
SSRY-64	0.58	66.00	5.00	0.58	0.38	0.52
SSRY-69	0.42	71.00	6.00	0.70	0.77	0.65
SSRY-78	0.69	65.00	5.00	0.48	0.35	0.43
SSRY-82	0.61	57.00	3.00	0.50	0.49	0.40
SSRY-103	0.53	73.00	6.00	0.54	0.86	0.44
SSRY-106	0.66	72.00	5.00	0.50	0.46	0.45
SSRY-120	0.67	9.00	3.00	0.49	0.22	0.44
SSRY-135	0.76	70.00	3.00	0.38	0.37	0.33
SSRY-147	0.95	74.00	3.00	0.10	0.07	0.10
SSRY-148	0.93	83.00	2.00	0.12	0.11	0.12
SSRY-151	0.45	73.00	8.00	0.70	0.75	0.66
SSRY-155	0.78	81.00	5.00	0.37	0.38	0.34
SSRY-161	0.59	81.00	6.00	0.61	0.54	0.57
SSRY-164	0.32	77.00	10.00	0.81	0.61	0.78
SSRY-169	0.88	86.00	5.00	0.22	0.10	0.21
SSRY-175	0.45	78.00	4.00	0.63	0.53	0.55
SSRY-177	0.49	70.00	4.00	0.57	0.36	0.48
SSRY-180	0.51	69.00	5.00	0.61	0.97	0.55
SSRY-181	0.63	63.00	3.00	0.54	0.62	0.48
SSRY-182	0.57	50.00	5.00	0.61	0.86	0.56
**Mean**	**0.62**	**61.29**	**4.77**	**0.55**	**0.43**	**0.45**
**SE**	**0.17**	**13.42**	**1.61**	**0.19**	**0.24**	**0.16**

The bold numbers presented on the row labelled Mean represents the mean values for the various marker details such as Allele frequency, nuumber of polymorphic bands, allele number per locus, gene diversity, etc. this information was used to determine which molecular marker had above average allele frequency among the lot chosen for the study. The S.E. represents the standard error for the various marker details and were used to compare which marker was more informative and whether significant differences existed among the molecular markers. H_o_ = Observed heterozygosity, PIC = Polymorphism information content, SE = Standard Error.

### 3.2 Within and between populations diversity

The analysis of molecular variance (AMOVA) based on the molecular data indicated higher within group variation which accounted for 97% of the total variation compared with variation between groups which accounted for only 3% of the total variation (Table 4). Comparatively, the local accessions had the highest PIC (0.44) and were also found to be the most diverse with the highest observed heterozygosity, allele number per locus and gene diversity (Table 5). Allele frequencies recorded were 0.71, 0.65 and 0.62 for accessions from CIAT, IITA and Local collections, respectively. Allele number per locus observed were 3.97, 3.91 and 2.69 for Local, IITA and CIAT accessions, respectively. Similarly, gene diversity recorded were 0.49, 0.47 and 0.40 respectively for Local, IITA and CIAT accessions.

**Table 4:** Analysis of molecular variance (AMOVA) of 89 cassava accessions from IITA, CIAT and Local sources.

Source of variation	DF	SS	MS	EV	% variation	Stat	Value	Probability^1^
Among population	2	71.36	35.68	0.649	3			
Within population	6	1559.89	18.13	18.138	97			
Total	88	1631.25		18.788	100	PhiPT	0.035	0.001

DF = Degrees of freedom, SS = Sum of squares, MS = Mean squares, EV = Estimated variance, *, *** = Significant at 5% and 1% probability respectively.

1 The probability is based on permutation across the full data set. PhiPT is a statistic measure for comparison between co-dominant data sets.

**Table 5:** Genetic diversity parameters among three populations of 89 cassava accessions from Ghana, IITA and CIAT.

Population	Sample size	Allele frequency	No. of polymorphic bands	Allele number per locus	Gene diversity	H_o_	PIC
IITA	40	0.65	27.09	3.91	0.47	0.42	0.42
CIAT	12	0.71	8.97	2.69	0.40	0.37	0.35
Local	37	0.62	25.23	3.97	0.49	0.44	0.44
Mean		0.65	30.65	3.83	0.47	0.42	0.42

IITA = Accessions from the International Institute of Tropical Agriculture, CIAT = accessions from the International Centre for Tropical Agriculture, Local = accessions obtained from local sources in Ghana, H_o_ = Observed heterozygosity, PIC = Polymorphism information content.

### 3.3 Population structure analysis

**Figure 1:**
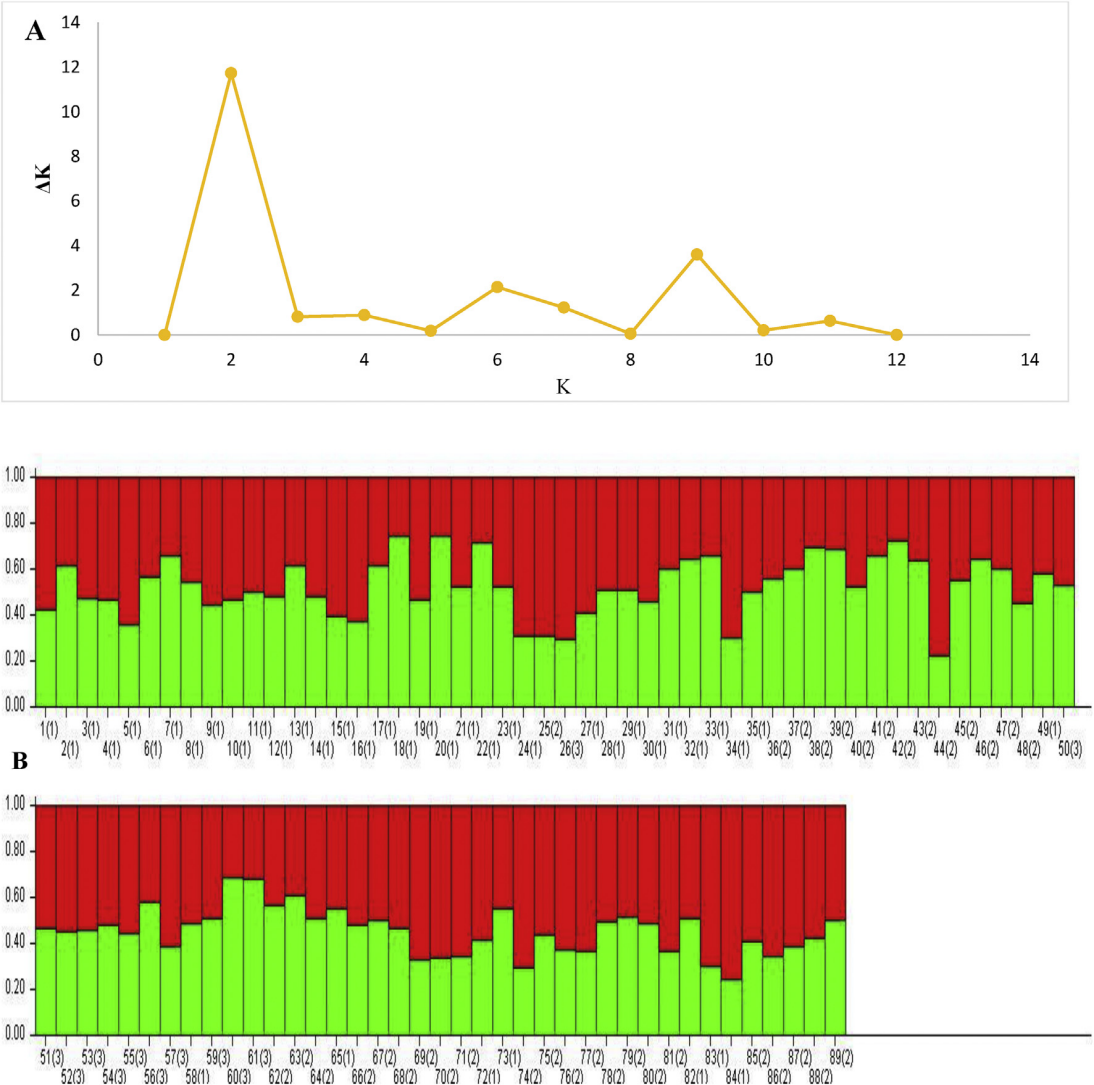
A: Delta K (ΔK) values for different numbers of populations assumed (K) in the STRUCURE analysis. B: Population structure of 89 cassava accession showing 2 sub population (Red indicates population 1 and Green population 2), each single line represents an accession. Population ID: 1 = IITA accessions, 2 = Local accessions, 3 = CIAT accessions.

The population structure analysis of the 89 cassava accessions estimated that the optimum number of subpopulation K which best explained the structure of the accessions was 2 (K = 2) using the Evanno method (Figure 1). Majority of the accessions (62) were classified as admixtures at a probability of association of ≥60%. The rest fell into two subpopulations. Subpopulation one consisted of 14 accessions and was made up of nine accessions from Local sources, four accessions from IITA and one accession from CIAT (Table 6). Subpopulation two on the other hand had 13 accessions which included six local accessions, five from IITA and two from CIAT. The 62 admixtures consisted of 31 accessions from IITA, 22 local accessions and nine accessions from CIAT. The allele frequency divergence observed among the two subpopulations (1 and 2) was 0.0505 with observed heterozygosity of 0.5857 for the more heterogeneous subpopulation one and 0.3849 for subpopulation two. However, subpopulation two had a higher fixation index (0.3772) indicating a high genetic diversity. Subpopulation one, on the other hand, showed very low genetic diversity with a fixation index of 0.0008.

**Figure 2:**
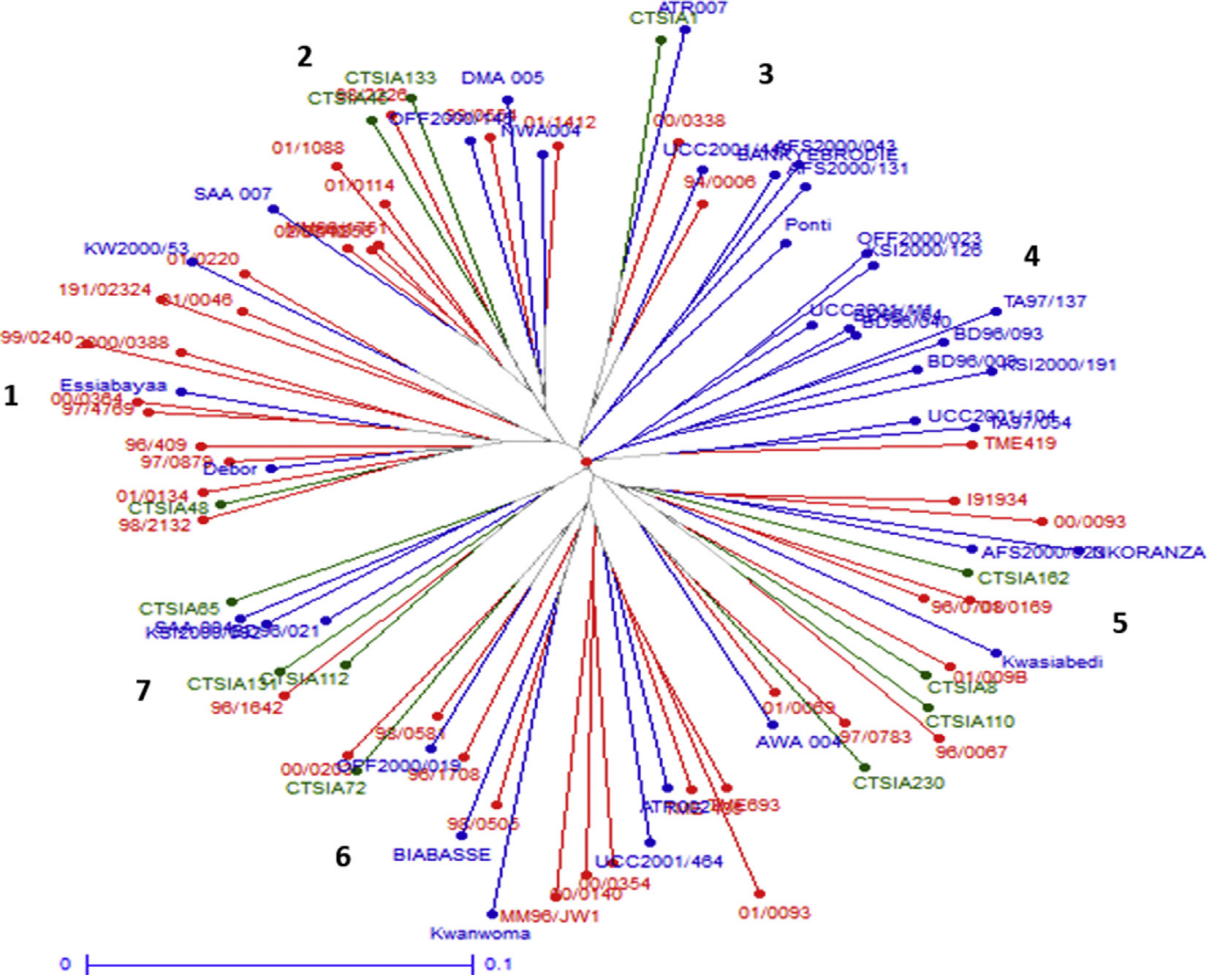
Dendrogram showing the relationship between 89 cassava accessions from Local (Blue), IITA (Red) and CIAT (Green) based on neighbour-joining analysis and simple matching coefficient.

Cluster analysis based on simple matching coefficient and neighbour-joining algorithim revealed seven ‘distinct’ clusters or sub-groupings with no apparent connections to the place of origin or collection of accessions (Figure 2). Specifically, cluster one includes 15 accessions, mainly dominated by accessions from IITA with only three local accessions (KW2000/53, Essiabayaa and Debor) and one from CIAT (CTSIA 48). Cluster two included 14 accessions from Local collections, IITA and CIAT. Cluster three included nine accessions: one from CIAT, two from IITA and six from Local collections. Out of the 12 genotypes in cluster four, only one came from IITA (TME 419) with no genotype from CIAT appearing in this cluster. Cluster five had 16 genotypes: three from Local collections, four from IITA and nine from CIAT. Cluster six which had a total of 16 accessions was dominated by accessions from IITA with only one genotype from CIAT. There were seven accessions in cluster seven: one from IITA, three from Local collections and three from the CIAT collections.

## 4 Discussion

The success of any crop breeding programme depends on the amount of genetic variability within the targeted traits for improvement and the extent to which these traits are heritable [49]. For this reason, assessment of genetic variability to aid parental line selections becomes a very important pre-breeding operation. Assessment of diversity at the molecular level can detect variations and/or markers linked to certain genomic regions, which is very reliable due to little or no environmental influence compared to phenotypic markers [50]. The level of genetic diversity is very important for the success or progress in breeding programmes [32]. The results of the current study reveal moderate to high levels of polymorphism across the 89 cassava accessions analysed, as was found for the genus *Manihot* at large [51]. The population structure analysis indicated two subpopulations and a large number of admixtures confirming the heterogenous nature of the cassava population used.

The average number of primers required to adequately assess genetic diversity in crops differ and depend on the level of out-crossing within the species [52]. Crop species that are highly inbred require larger number of primers than crops that are naturally heterogeneous, like cassava. The average number of 4.77 alleles per locus detected by the SSR primers (Table 3) is similar to earlier findings on genetic diversity analyses in cassava [40, 53] indicating that the SSR markers used in the current analysis were informative and appeared to be sufficient for the assessment of genetic diversity in the 89 cassava genotypes.

The observed heterozygosity levels (0.03–0.97, mean = 0.43) were higher than those found for 64 accessions of cassava with 26 SSR markers, where the heterozygosity levels ranged from 0.47 to 0.66 with a mean of 0.57 [40]. These lower levels of heterozygosity reported could be due to the lower numbers of both accessions and SSR markers used as compared to this study. The high level of heterozygosity observed within the accessions here could be attributed to three reasons. Firstly, the diverse geographical background (place of collection) of the accessions used in the current study. Specifically, the collections obtained from IITA could likely be made up of accessions originating from various geographical areas (countries) in West Africa. Likewise, the accessions obtained from CIAT are more likely to be originated from diverse geographical origins (countries) within the Latin American region. Secondly, there could be ‘secondary’ mixing of the gene pool from the CIAT and IITA collections through exchange of genetic materials and hybridization and thirdly, the materials collected from the farmers' field could be part of introductions by research and extension staff through on-farm demonstrations. The genetic similarity among most of the groups could be a reflection of the exchange of genetic materials among farmers, resulting in the generation of duplicates [37, 54]. Accessions collected from farmers fields need to be characterized to remove duplicates for effective breeding.

Cassava is naturally highly heterozygous and diploid (2n = 36) with some few rare cases of polyploidy (2n = 3x = 54 and 2n = 108) being recorded [55]. But varieties are cloned and although heterozygous, they are uniform. The results from the analysis of molecular variance indicated greater genetic variation within the populations than among populations which is similar to earlier studies on within and between population genetic variation in cassava genotypes from different sources [40, 56, 57]. The lower level of variation between populations compared with within population variation could be due to selection by farmers for similar traits at the different collection sources. However, within a particular germplasm collection, farmers keep diverse genotypes. Among the accessions from the different sources, the Local accessions had the highest number of alleles per locus, gene diversity, observed heterozygosity and PIC, suggesting that farmers normally keep mixtures of cassava varieties for diverse utilisation purposes and to guard against total crop failure. The implication of this to cassava breeding in Ghana is that, ‘farmer held accessions’ remain valuable sources of novel traits/genes for the genetic improvement of cassava in the country. Local genetic materials serve as rich sources of genetic diversity which can be used to complement and broaden the gene pool of advanced accessions [58]. The detected genetic diversity in the landrace population if phenotypically linked with traits of economic importance can be exploited for further enhancement of the germplasm [11, 12]. Notwithstanding, the population structure analysis indicated that a large proportion of the accessions from IITA were admixtures (Table 6), indicating the possibility of sharing common ancestory with most of the accessions from the other sources.

**Table 6:** Inferred subpopulation of the accessions showing their probabilities of association to each sub population based on fixation index.

Accessions	Pop ID	Populations		Inferred subpopulation	Accessions	Pop ID	Populations		Inferred subpopulation
		1	2				1	2	
00/0354	1	0.604	0.396	1	BIABASSE	2	0.420	0.580	Admixture
96/1708	1	0.637	0.363	1	96/409	1	0.447	0.553	Admixture
DMA 005	2	0.653	0.347	1	BD96/093	2	0.444	0.556	Admixture
CTSIA 48	3	0.654	0.346	1	Debor	2	0.448	0.552	Admixture
99/0240	1	0.658	0.342	1	Essiabayaa	2	0.420	0.580	Admixture
BD 96/040	2	0.710	0.290	1	00/0203	1	0.503	0.497	Admixture
KW 2000/53	2	0.627	0.373	1	00/0338	1	0.518	0.482	Admixture
Kwanwoma	2	0.606	0.394	1	01/0069	1	0.458	0.542	Admixture
Nkoranza	2	0.616	0.384	1	01/0093	1	0.537	0.463	Admixture
SAA 004	2	0.659	0.341	1	TME435	1	0.540	0.460	Admixture
OFF2000/019	2	0.607	0.393	1	01/0114	1	0.495	0.505	Admixture
Kwasiabedi	2	0.633	0.367	1	01/0134	1	0.517	0.483	Admixture
TME693	1	0.709	0.291	1	01/0220	1	0.508	0.492	Admixture
UCC2001/111	2	0.605	0.395	1	2000/0388	1	0.529	0.471	Admixture
01/0046	1	0.381	0.619	2	96/0067	1	0.471	0.529	Admixture
191/02324	1	0.318	0.682	2	96/1642	1	0.468	0.532	Admixture
94/0006	1	0.317	0.683	2	97/1856	1	0.507	0.493	Admixture
96/0708	1	0.325	0.675	2	97/4769	1	0.497	0.503	Admixture
98/2226	1	0.397	0.603	2	98/0505	1	0.541	0.459	Admixture
AFS2000/131	2	0.369	0.631	2	99/0554	1	0.515	0.485	Admixture
ATR002	2	0.347	0.653	2	AFS2000/023	2	0.469	0.531	Admixture
Bankyebrodie	2	0.392	0.608	2	ATR007	2	0.478	0.522	Admixture
BD96/009	2	0.340	0.660	2	SAA007	2	0.532	0.468	Admixture
BD96/021	2	0.398	0.602	2	CTSIA1	3	0.460	0.540	Admixture
BD96/154	2	0.390	0.610	2	CTSIA110	3	0.509	0.491	Admixture
CTSIA72	3	0.354	0.646	2	CTSIA112	3	0.511	0.489	Admixture
CTSIA8	3	0.360	0.640	2	CTSIA131	3	0.527	0.473	Admixture
00/0093	1	0.555	0.445	Admixture	CTSIA133	3	0.496	0.504	Admixture
01/1088	1	0.577	0.423	Admixture	CTSIA65	3	0.538	0.462	Admixture
01/1412	1	0.578	0.422	Admixture	CTSIA230	3	0.457	0.543	Admixture
97/0879	1	0.552	0.448	Admixture	97/0783	1	0.498	0.502	Admixture
CTSIA 45	3	0.589	0.411	Admixture	CTSIA162	3	0.489	0.511	Admixture
01/0169	1	0.569	0.431	Admixture	NWA004	2	0.490	0.510	Admixture
OFF2000/023	2	0.592	0.408	Admixture	I91934	1	0.471	0.529	Admixture
TA97/137	2	0.584	0.416	Admixture	KSI2000/092	2	0.504	0.496	Admixture
UCC2001/104	2	0.581	0.419	Admixture	KSI2000/126	2	0.482	0.518	Admixture
UCC2001/449	2	0.571	0.429	Admixture	KSI2000/191	2	0.520	0.480	Admixture
UCC2001/464	2	0.562	0.438	Admixture	MM96/JW1	1	0.461	0.539	Admixture
00/0140	1	0.421	0.579	Admixture	BAN 001	2	0.539	0.461	Admixture
00/0364	1	0.447	0.553	Admixture	OFF2000/145	2	0.497	0.503	Admixture
MM96/1751	1	0.423	0.577	Admixture	Ponti	2	0.484	0.516	Admixture
02/0540	1	0.401	0.599	Admixture	TA97/054	2	0.498	0.502	Admixture
98/0581	1	0.434	0.566	Admixture	TME419	1	0.481	0.519	Admixture
98/2132	1	0.404	0.596	Admixture	AWA004	2	0.542	0.458	Admixture
AFS2000/043	2	0.430	0.570	Admixture					

Population ID: 1 = IITA accessions, 2 = Local accessions, 3 = CIAT accessions.

Precise identification of phylogenic relationship and divergence of germplasm population(s) gives very useful information which helps decision making (especially choice of parental lines for hybridization schemes) in breeding programmes [59]. The results of the current study separated the 89 cassava accessions into two broad subpopulations and admixtures according to the STRUCTURE analysis and, further, into seven clusters based on the neighbour-joining algorithim. The clustering of the 89 cassava accessions into sub-groups or clusters apparently had little or no connection to the place of collection (geographical origin) of the accessions. Apart from cluster four (Figure 2), all clusters contained at least one or more accessions from the Local, IITA or CIAT collections. This grouping of genotypes into clusters irrespective of geographical origins is similar to an earlier study of cassava accessions [5]. The apparent lack of impact of the place of origin on the population structure in the current study, suggests the possibility that most or all of the accessions in the current study have syntenic relations (presence of common alleles across the accessions) [57, 60]. As discussed earlier, this relationship could include factors such as movement of improved varieties to farmer's fields during participatory breeding programs, germplasm collection from farmers' fields, and accessions which are progenies of ‘secondary’ hybridization of parents from different sources [57].

The presence of a large proportion of admixtures among the accessions confirms the heterogenous nature of cassava, being predominantly an outcrossing crop. However, larger proportion of admixtures was found among the IITA accessions compared with the Local collections and those from CIAT, which indicates that most of the accessions collected from IITA might share ancestors in earlier collections from several parts of Africa including Ghana. The population structure observed in the current study indicates the existence of similarities among accessions from different origins suggesting that it will be more useful to select parents based on genetic relatedness rather than based on their origins alone.

## 5 Conclusion

The simple sequence repeat markers used were informative enough and could detect genetic diversity among the cassava accessions to warrant selection. Greater genetic variation was detected within population than among populations. The Local accessions were more genetically diverse compared to those obtained from IITA and CIAT. The population structure of the cassava accessions did not show any apparent link to geographical origin (place of collection). Farmer-held germplasm accessions of cassava remain important sources of rich genetic resources for cassava improvement.

## Declarations

### Author contribution statement

Joseph Adjebeng-Danquah: Conceived and designed the experiments; performed the experiment; contributed reagents, materials, analysis tools or data, analysed and interpreted the data; wrote the paper.

Joseph Manu-Aduening: Conceived and designed the experiments; analysed and interpreted the data; wrote the paper.

Isaac Kwadwo Asante: Conceived and designed the experiments, wrote the paper.

Richard Yaw Agyare: Contributed reagents, materials, analysis tools or data; wrote the paper.

Vernon Gracen: Conceived and designed the experiments; wrote the paper.

Samuel Kwame Offei: Conceived and designed the experiments; wrote the paper.

### Funding statement

J. Adjebeng-Danquah was supported by the West Africa Centre for Crop Improvement (WACCI) PhD program, which received funding from The Generation Challenge Programme (GCP) and the Alliance for a Green Revolution in Africa (AGRA).

### Competing interest statement

The authors declare no conflict of interest.

### Additional information

No additional information is available for this paper.
